# Fully amino acid-based hydrogel as potential scaffold for cell culturing and drug delivery

**DOI:** 10.3762/bjnano.10.249

**Published:** 2019-12-27

**Authors:** Dávid Juriga, Evelin Sipos, Orsolya Hegedűs, Gábor Varga, Miklós Zrínyi, Krisztina S Nagy, Angéla Jedlovszky-Hajdú

**Affiliations:** 1 Laboratory of Nanochemistry, Department of Biophysics and Radiation Biology, Semmelweis University, Nagyvarad square 4, Budapest, Hungary; 2 Department of Oral Biology, Semmelweis University, Nagyvarad square 4, Budapest, Hungary

**Keywords:** biocompatibility, cystamine, hydrogel, lysine, poly(amino acid), poly(aspartic acid), polymer

## Abstract

Polymer hydrogels are ideal scaffolds for both tissue engineering and drug delivery. A great advantage of poly(amino acid)-based hydrogels is their high similarity to natural proteins. However, their expensive and complicated synthesis often limits their application. The use of poly(aspartic acid) (PASP) seems an appropriate solution for this problem due to the relatively cheap and simple synthesis of PASP. Using amino acids not only as building blocks in the polymer backbone but also as cross-linkers can improve the biocompatibility and the biodegradability of the hydrogel. In this paper, PASP cross-linked with cystamine (CYS) and lysine-methylester (LYS) was introduced as fully amino acid-based polymer hydrogel. Gels were synthesized employing six different ratios of CYS and LYS. The pH dependent swelling degree and the concentration of the elastically active chain were determined. After reduction of the disulfide bonds of CYS, the presence of thiol side groups was also detected. To determine the concentration of the reactive cross-linkers in the hydrogels, a new method based on the examination of the swelling behavior was established. Using metoprolol as a model drug, cell proliferation and drug release kinetics were studied at different LYS contents and in the presence of thiol groups. The optimal ratio of cross-linkers for the proliferation of periodontal ligament cells was found to be 60−80% LYS and 20−40% CYS. The reductive conditions resulted in an increased drug release due to the cleavage of disulfide bridges in the hydrogels. Consequently, these hydrogels provide new possibilities in the fields of both tissue engineering and controlled drug delivery.

## Introduction

The number of medical applications of polymer hydrogels increased substantially during the last decades due to their similarity to soft tissues [1–3]. Polymer hydrogels possess properties of solid materials such as deformability and rigidity, but at the same time they are permeable for small molecules. One of the most beneficial properties of polymer hydrogels is that they are sensitive to alterations of the local environmental conditions, such as the pH value or temperature. Polymer hydrogels can change their swelling degree depending on the changing environmental conditions. Therefore, hydrogels can be used as drug delivery systems [4], implants [5–6], coatings [7–8] or scaffolds for tissue engineering [2–3,9–10]. Besides these stimuli-responsive properties, the chemical and physical structure, the mechanical properties [10] as well as biocompatibility and biodegradability are also fundamental features of polymers developed for numerous medical applications [11–12].

Polymer hydrogels are capable of releasing physically entrapped drug molecules. The release kinetics of the loaded drug molecules depend significantly on the swelling degree of the polymer matrix [13–16]. Recently, smart hydrogels showing strong and abrupt responses (changes in their swelling degree) to small changes of the environmental conditions have been the subject of extensive research [17–22].

The composition of the polymer backbone determines almost all of the properties of the hydrogels. For biological applications, in general, the polymer backbone and its degradation products have to be biodegradable and biocompatible [11,23]. In contrast to many synthetic polymers, the degradation products of poly(amino acid)s, which are mainly built from only one or two types of amino acids, are biocompatible nutrients. In addition, poly(amino acid)s have enormous structural diversity and they supposedly lack immunogenicity [24–25]. In summary, the use of poly(amino acid)s has practically only one disadvantage: their synthesis is usually complicated and expensive [3].

Recently, various types of polymer-based hydrogels have been developed for purposes of tissue engineering and regenerative medicine [26]. Most of these polymers try to mimic or recreate the natural environment of the cells, namely the extracellular matrix (ECM) [27]. Among them, synthetic, amino acid-based polymers attract particular attention due to their tunable properties and the structural similarity to the native ECM [26]. The poly(amino acid)s being tested for biomedical applicability can be classified into three groups: anionic, cationic and neutral poly(amino acid)s [28–29]. The effect of poly-ʟ-lysine, which has a cationic character, on the cell behavior has been widely studied in the field of tissue engineering [30–31], while among the anionic poly(amino acid)s, poly(glutamic acid) is typically used for the development of hydrogel scaffolds [32]. Moreover, our research group demonstrated previously that hydrogels based on the anionic poly(aspartic acid) (PASP) are also well-suited for tissue engineering purposes [25].

Another field of potential biomedical applications of poly(amino acid)s is drug delivery. Poly(amino acid)-based microcarriers can improve the pharmacological and therapeutic properties of various drugs [28]. By applying such microcarriers, the drug release kinetics can be controlled. Among the anionic amino acids, glutamic acid was previously used for the preparation of polymer-based microcarriers [33]. However, there are only sporadic data available regarding PASP-based drug delivery systems [34–37].

Although the preparation of most types of poly(amino acid)s is expensive, the synthesis of PASP can be relatively cost-efficient, and it does not require extreme conditions, as we described previously [25,37–40]. In the last decades, the interest of scientists in PASP-based hydrogels increased significantly due to their potential applications in medical, pharmaceutical and environmental fields [25,34,41–44]. Several publications can be found concerning different types of cross-linkers to prepare PASP-based hydrogels [22,34,45]. However, the number of studies describing the use of pure amino acids or amino acid derivatives as cross-linkers is limited [22,46]. For hydrogels made of fully amino acid-based polymers, biodegradability as well as biocompatibility could be improved due to their specific chemical composition. In our previous publications, we presented the synthesis of PASP and several preparation methods of PASP-based hydrogels based on cross-linking with diaminobutane (DAB) and CYS in two-step reactions [21–22,25]. The chemical [22], swelling [13], mechanical and responsive properties [21] of these gels as well as their applicability as scaffolds for osteosarcoma cells (MG-63) [25] have been investigated. We showed that MG-63 cells can proliferate on PASP-based hydrogels [25], which led to the idea that similar fully amino acid-based gels may also support the growth of untransformed cells.

In the present study, derivatives based exclusively on amino acids, namely, on lysine-methylester (LYS) and CYS, were used as cross-linkers for preparing PASP-based hydrogels. CYS is biologically active and can provide free thiol groups under reductive conditions, while the advantage of LYS is that it can partially decrease the anionic character of aspartic acid. In addition, LYS also facilitates the electrostatic interaction between anionic plasma membrane sites and cationic polymer sites. Therefore, it supports cell adhesion and proliferation [47]. The swelling, mechanical and degradation properties of the gels containing LYS and CYS at different ratios were investigated since these attributes are crucial for biomedical applications. The swelling degree was determined at different pH values and under redox conditions. For biocompatibility studies, cell viability tests were carried out using primary cultures of human periodontal ligament-derived cells (PDLCs) instead of the previously used tumor cell line. Periodontal ligament as a source of stem-like cells is easily accessible during surgical removal of wisdom teeth [48]. PDLCs hold great promise for application in the field of tissue engineering [49]. Cell viability was assessed using the WST-1 reagent [2-(4-iodophenyl)-3-(4-nitrophenyl)-5-(2,4-disulfophenyl)-2*H*-tetrazolium] (Roche, Switzerland), while the cell morphology was observed under a two-photon microscope. Moreover, the release kinetics of the model drug metoprolol were also examined under various environmental conditions.

## Experimental

### Materials

Following materials have been used in this study: ʟ-aspartic acid (CAS: 56-84-8, Aldrich, ≥98%), orthophosphoric acid (CAS: 7664-38-2, Aldrich, ≥99%), dimethylformamide (CAS: 68-12-2, VWR, ≥99.9%) cystamine dihydrochloride (CAS: 56-17-7, Fluka, ≥98%), lysine methyl ester dihydrochloride (CAS: 26348-70-9, Bachem, 97%), dimethyl sulfoxide (CAS: 67-68-5, VWR, ≥99%), dibutylamine (CAS: 111-92-2, Aldrich, ≥99.5%), citric acid monohydrate (CAS: 5949-29-1, VWR, 100%, normapur), imidazole (CAS.288-32-4, Sigma-Aldrich, ≥99.5%, puriss), sodium chloride (CAS: 7647-14-5, Sigma-Aldrich, puriss), sodium hydroxide (CAS: 1310-73-2, Reanal, puriss), borax (CAS: 1303-96-4, Hungaropharma, ≥99.5%), disodium hydrogen phosphate (CAS: 7558-79-4, Sigma-Aldrich, ≥98%), trisodium phosphate (CAS: 10101-89-0, Sigma-Aldrich, ≥98%), phosphate-buffered saline (PBS) tablet (Sigma), ᴅʟ-dithiothreitol (CAS: 3483-12-3, Sigma, ≥99%).

### Synthesis of poly(succinimide)

Poly(succinimide) (PSI) was synthesized by thermal polycondensation of the mixture of ʟ-aspartic acid and orthophosphoric acid. The mixture was loaded into a 1 L flask and inserted into a rotary evaporator (IKA). The temperature was increased to 180 °C while the pressure was decreased to 5 mbar. After 7 h, brown foam was gained, which was dissolved in dimethylformamide (DMF). Subsequently, the solution was precipitated, washed with distilled water and finally dried at 40 °C. Details of this method were published previously [25,37–38].

### Simultaneous cross-linking of PSI by CYS and LYS

**Figure 1:**
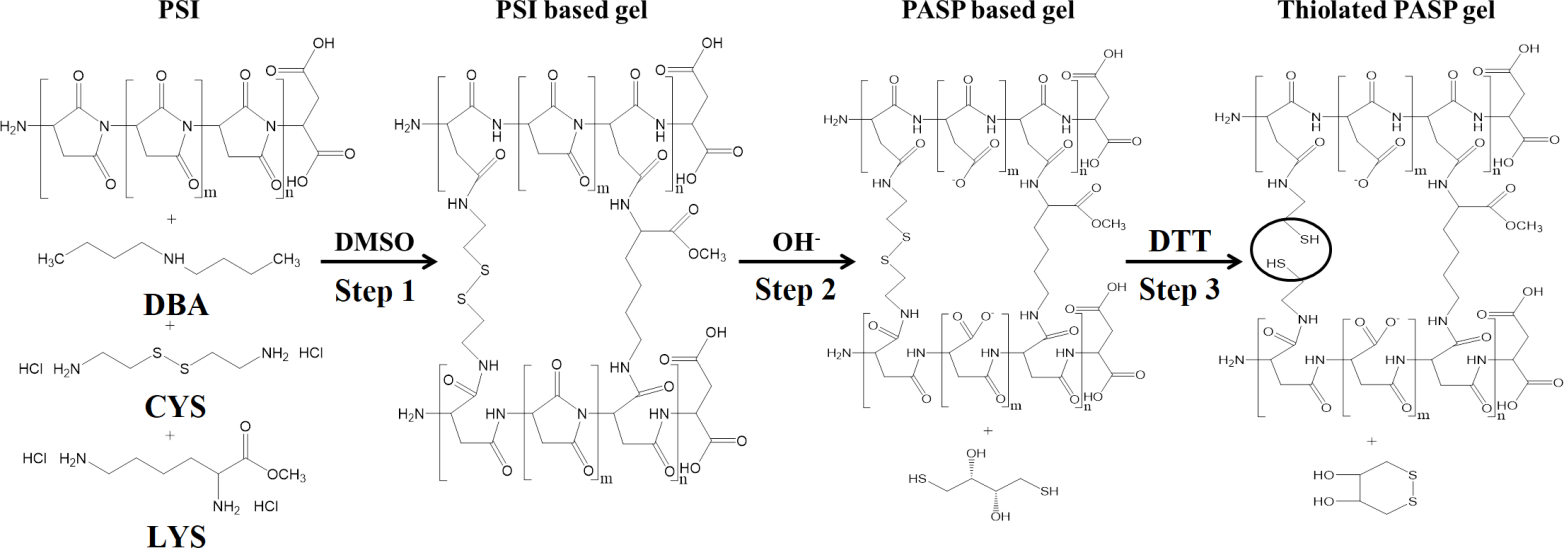
Synthesis of the simultaneously cross-linked and thiolated PSI and PASP gels.

PSI was dissolved in dimethyl sulfoxide (DMSO) at a concentration of 25 wt % and mixed with the cross-linker solution, which contained cystamine dihydrochloride (CYS·2HCl) and lysine methyl ester dihydrochloride (LYS·2HCl) at different ratios in DMSO (Table 1). During cross-linking, the free primary amino groups of the cross-linkers react with the imide groups of the succinimide rings in a nucleophilic addition reaction (Figure 1, step 1). Dibutylamine (DBA) was added to the reaction mixture in order to adjust the pH value.

**Table 1:** The applied amounts of the various components during the gel synthesis.

Gel sample	PSI solution (mg)	CYS·2HCl (mg)	LYS·2HCl (mg)	DMSO (μL)	DBA (μL)

PSI-100CYS-LYS	600	17.4	0.0	325.5	26.1
PSI-80CYS-LYS	600	13.9	3.6	325.4	26.1
PSI-60CYS-LYS	600	10.4	7.2	325.3	26.1
PSI-40CYS-LYS	600	7.0	10.8	325.1	26.1
PSI-20CYS-LYS	600	3.5	14.4	325.0	26.1
PSI-0CYS-LYS	600	0.0	18.0	324.9	26.1

The mixture was loaded into glass frames in order to prepare gel films. After 24 h, the gelation occurred and every sample had a PSI content of 15 wt % at a theoretical degree of 20 cross-links. The degree of cross-links is defined as the molar ratio of monomers and cross-linking agents. The reaction of the gel formation is shown in Figure 1, step 1.

Six gel types were prepared with different ratio of the cross-linking agents (Table 1). The number in the name of the samples indicates the molar percentage of CYS of the total amount of cross-linking agents. For example, PSI-100CYS-LYS contains only CYS while in the case of PSI-0CYS-LYS, only LYS was used as cross-linking agent.

### Preparation of PASP-based hydrogels

PASP-based hydrogels were formed by mild alkaline hydrolysis of the PSI gels. The gels were immersed into an imidazole-based buffer (pH 8, concentration *c* = 0.1 M, ionic strength *I* = 0.25 M). The buffer was changed daily for four days. The reaction is depicted in Figure 1, step 2.

To prepare thiolated PASP-based hydrogels, 0.1 M dithiothreitol (DTT) solution was used, prepared with the same imidazole buffer that was mentioned previously. The cleavage of the disulfide bonds leads to the formation of cysteamine molecules in the polymer matrix, which is indicated by CYSE instead of CYS in the nomenclature. The reaction is shown in Figure 1, step 3.

### Effect of the pH value on the equilibrium degree of swelling

To determine the pH value-dependent degree of swelling of both the PSI- and the PASP-based gels, gel disks of three different diameters (6, 10 and 14 mm) were prepared. The weight of the disks was measured after reaching the equilibrium swelling degree in DMSO (for the PSI-based gels) or in an imidazole buffer of pH 8 (for the PASP-based gels). After that, the gel disks were immersed in buffers with different pH values (2.3–13.8) but the same ionic strength (*c* = 0.1 M, *I* = 0.25 M) for two days. Then, the buffers were changed, and the weight of the disks was measured two days later, following that the liquid had been carefully sponged up from the outer surface of the gels. Finally, the gels were dried at 40 °C, and the degree of swelling was calculated as the quotient of the weight of the swelled and the dried gel (*m*_dried_). Three parallel measurements were carried out for each gel diameter.

### Determination of the concentration of the elastically active chains by mechanical measurements

To measure the elastic modulus (*G*), gel cylinders of 1 cm in diameter and height were prepared with the same constitution as described above. *G* was assessed by vertical stress–strain measurements using an Instron 5942 (Intest kft., Hungary) mechanical tester, and its exact value was calculated by determining the stress–strain behavior during unidirectional compression. The temperature and the volume of the gels were constant during deformation, therefore, the Neo–Hooken law could be used to describe the deformation of the gels [21]:

[1] $$\sigma_{\text{N}}=-G(\lambda -\lambda^{\left(-2\right)})$$

where σ_N_ is the nominal stress and λ is the deformation ratio, which can be calculated as the ratio of the actual height and the initial height of the cylinder. *G* can be expressed as:

[2] $$G=RTA\cdot v^{*}\cdot q_{0}{}^{-2/3}\cdot \Phi^{1/3}$$

where *R* is the gas constant, *T* is the temperature, Φ represents the polymer volume fraction of the gel, v* is the concentration of the elastically active chains in the dry network (expressed in moles), and *q*_0_ denotes the so-called memory term, by which the network “remembers” its initial state. The molecular interpretation of *q*_0_ is controversial for networks prepared in solution. *A* is a model-dependent parameter. According to the Flory theory *A* = 1, while in the James–Guth and Staverman theories *A* = 0.5 [50–51]. For the present experiments, the parameter of the Flory theory was used. The value of ν* was calculated using Equation 2.

### Swelling of the PASP hydrogels in DTT solution

Different PASP-XCYS-LYS gel disks of 6 mm in diameter were prepared and immersed in a 0.1 M DTT solution at pH 8. Changes of the concentration were avoided during the measurements by using a large amount of the solution in relation to the gel disks. The swelling kinetics were monitored under an Alpha ScopeTek STO-3 light microscope. Pictures were taken every 5 min during one day and the ScopePhoto program was used to measure the diameter of the gel disks. In each case, three parallel measurements were carried out. The relative degree of swelling (*Q*_rel_) was calculated as a ratio of the volume of the gels (*Q*_V_) after and before the DTT treatment.

[3] $$Q_{\text{rel}}=\frac{Q_{\text{V}\text{, after DTT}}}{Q_{\text{V}\text{, before DTT}}}$$

To study the effect of the DTT concentration on the swelling degree of the gels, PASP-20CYS-LYS gels were immersed in DTT solutions of pH 8 at different DTT concentrations (0.001−1.6 mmol/mL). In order to avoid the oxidation of DTT, the samples were rinsed with nitrogen gas. The weight of the gel disks was measured before and after being treated with DTT over five days. For all samples, two parallel measurements were carried out. The kinetic evaluation is based on the Tanaka–Fillmore–Peters–Candau (TFPC) theory [13].

### Isolation and culturing of tooth-derived cells

The PDLCs originated from human wisdom teeth, which were surgically removed from healthy young adults at the Department of Dentoalveolar Surgery, Semmelweis University (according to the ethical guidelines set by the Ethical Committee of the Hungarian Medical Research Council). This study was approved by the Semmelweis University Regional and Institutional Committee of Science and Research Ethics. The number of the ethical permission is: 17458/2012/EKU. After extraction, the teeth were immediately placed into a sterile cell culture medium. The viable periodontal fibers were removed from the tooth surface, put into a sterile box with a sterile blade and digested in 1 mL collagenase I solution (1 mg/mL, Sigma-Aldrich, St. Louis, Missouri) for 1 h at 37 °C. The samples were vortexed every 10 min. After digestion, the fibers were mechanically loosened with needles (21G and 18G) and were centrifuged for 5 min at 250*g*.

The PDLCs were maintained in a humidified incubator (Nuaire, USA) in 100 mm tissue culture dishes (Orange Scientific, Belgium) under standard culture conditions (37 °C, 5% CO_2_, 100% humidity). The growth medium of the PDLCs was the following. Eagle’s Minimal Essential Medium Alfa (αMEM) (Gibco, USA) was supplemented with 10% fetal bovine serum (FBS, Gibco, USA), 2 mM ʟ-glutamine (Gibco, USA), 100 units/mL penicillin and 100 mg/mL streptomycin (Gibco, USA). When the cell culture became subconfluent, it was passaged at a ratio of 1:20 using a 0.05% trypsin/EDTA solution.

### Cell viability assay

Before performing the cell viability assay, the PASP-based gel disks of different constitutions were incubated in the above-mentioned growth medium for 3 h with a medium change after 1.5 h. After incubation, the gel disks were placed into low cell-binding plates (96-well plates, Nunc^TM^ Dishes, St.Louis, Missouri) and sterilized by UV radiation for 1 h. Subsequently, 20,000 PDLCs were seeded on each gel disk in 200 μL medium and cultured for one or three days. Afterwards, each well was washed with PBS (37 °C) to remove swimming or loosely attached cells. To measure the cell viability, a colorimetric assay was performed using the cell proliferation reagent WST-1. The reagent was diluted (1:20) with αMEM containing no phenol red (Gibco, USA). Then, 200 μL solution was applied in each well. After incubation for 2 h at 37 °C, 150 μL of the supernatant solution was transferred from each well into an empty 96-well plate. The absorbance was measured at 450 nm using a microplate reader (Model 3550, Bio-Rad Laboratories, Japan) with a reference wavelength of 655 nm. Gel disks without cells were used as background controls.

### Microscopic study of the cells

To visualize the PDLCs, the cells were labelled with a vital dye called Vybrant DiD (Molecular Probes, USA) before seeding. Gel disks of 5 mm diameter were placed into 48-well plates, and 40,000 cells per well were seeded in 400 μL medium. After 1 or 3 days, the wells were washed with PBS (37 °C) and fixed in 4% paraformaldehyde (in PBS) at room temperature for 2 h. The fixed samples were washed twice and then stored in PBS at 4 °C. The examination was carried out under a two-photon microscope (Femto2d, Femtonics, Hungary). A SpectraPhysics DeepSee laser was used at a wavelength of 800 nm to excite the photoactive dye. Images were taken with a 10x objective by the MES4.4v program. The cells can be seen in red color, while green indicates the autofluorescence of the PASP-based gels.

### Statistical analysis

To assess the cell viability, PDLC cultures derived from five different patients were used, and five parallel measurements were carried out for each culture. The arithmetic mean and the standard error of the mean (SEM) are indicated in the diagrams. The statistical evaluation of the data was performed by the STATISTICA 10 software applying the Kruskal−Wallis nonparametric ANOVA test followed by a median test. A derived difference was regarded as statistically significant if the probability value is smaller than 0.05 (*p* < 0.05).

### Drug release measurement

For the drug release measurement metoprolol was used as a model drug. Gel disks with a thickness of 0.75 mm and a diameter of 6 mm were swelled in physiological saline solution to reach the equilibrium swelling degree. After that, they were immersed in 100 mg/mL metoprolol tartrate (Sigma-Aldrich) solution for three days, and then the solution was carefully sponged up from the gel surface prior to the measurement. The release of the metoprolol was measured in 20 mL physiological saline solution (*c*_NaCl_ = 9 g/L, pH 5.5) and in 20 mL 0.1 M DTT (in physiological saline solution). The drug release was followed by a JASCO V-650 spectrophotometer at 274 nm with an optical fiber probe. The concentration of the released metoprolol was calculated using the calibration line (Supporting Information File 1, Figure S1). The mass of the gel disks was measured before the experiment. The concentration was divided by the corresponding mass of the gel to compensate for variations of the gel mass during the parallel measurements.

## Results and Discussion

### Influence of the pH value on the equilibrium degree of swelling of PSI and the PASP-XCYS-LYS hydrogels

**Figure 2:**
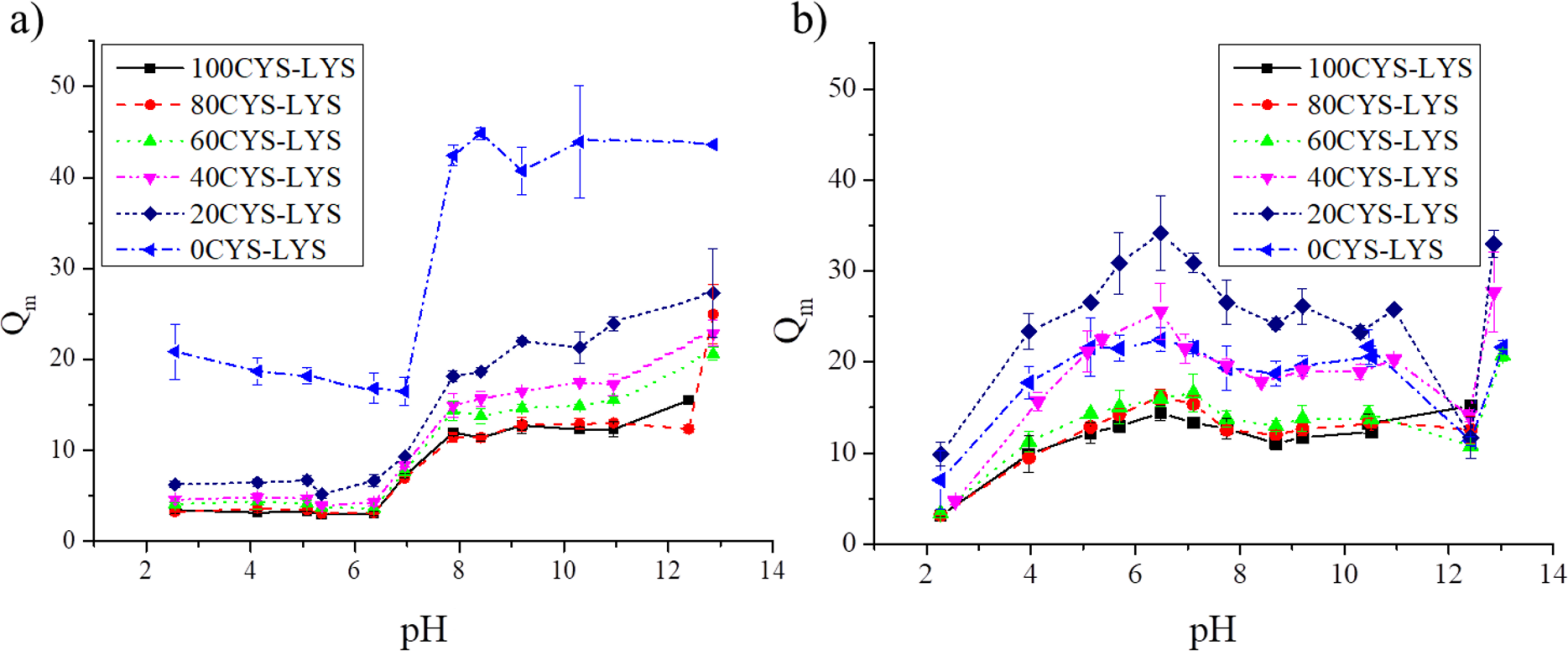
The dependence of the mass swelling degree of (a) the PSI- and (b) the PASP-based gels on the pH value.

For applications in the field of tissue engineering, the swelling behavior of the amino acid-based hydrogels needs to be determined. Ideally, the swelling properties of the hydrogel scaffold should not change during the application. However, different cell types require scaffolds with different swelling properties (and also different stiffness). The PSI-based gels show a low swelling degree at pH 8 due to the hydrophobic character of the PSI backbone (Figure 2a). Since PSI is insoluble in water, the Huggins interaction parameter increases in an aqueous medium, hence, the gels shrink. Between pH 6 and 8, the swelling degrees abruptly rise as the polymer networks are hydrolyzed. The successful hydrolysis was proved by FTIR spectroscopy (Supporting Information File 1, Figure S2). This kind of abrupt change was described in previous articles, where other molecules or only LYS were applied as cross-linkers [21–22,34]. Between pH 8 and 11, the swelling degrees remain approximately constant, as has been observed for other types of PASP-based hydrogels [21–22,34]. However, for pH values larger than 11, a further increase in the swelling degree is observed (Figure 2a). This increase is due to the cleavage of the disulfide bonds in CYS, as we described in our previous article for DAB and CYS cross-linked hydrogels [21]. Another reason for the swelling can be the hydrolysis of the ester bond in LYS, which increases the concentration of negatively charged groups in the polymer matrix. The PSI-0CYS-LYS gel shows a significantly higher swelling degree at every pH value due to the smaller reactivity of the amine groups in LYS, which leads to a lower amount of cross-links [21].

Regarding the mass swelling degree of the PASP-based hydrogels, two groups can be observed which differ in the ratio of the LYS and the CYS cross-linkers (Figure 2b). The gels with a lower ratio of LYS cross-links (100, 80 and 60CYS-LYS) have a lower swelling degree than the gels with higher LYS concentration (40, 20 and 0CYS-LYS) at every pH value, which also proves the lower reactivity of LYS. The highest swelling degree was measured around pH 6, except for PASP-100CYS-LYS, in which every carboxyl group on the side chains of the aspartic acid monomers is deprotonated. By simultaneously using DAB or DAB/CYS as cross-linking agents, the degree of swelling is increasingly sharp in the range of pH 3–5 [22]. However, the use of LYS causes a monotonic increase of the swelling degree in the acidic pH range due to the free amine groups in the LYS side chain. Protonation of the free amine groups in CYS and LYS results in a decrease of the swelling degree between pH 6 and 10. However, for the DAB cross-linker, the degree of swelling remained constant at alkaline pH values as we published in our previous paper [21–22,45]. The further increase of the swelling degree at pH values larger than 12 is the consequence of the cleavage of the disulfide bond of CYS and the hydrolysis of the ester group in the LYS cross-linkers. These findings are in a good agreement with our previous study. Yet, the LYS cross-linker shows a rather different dependence on the pH value than the DAB cross-linker, which could enhance the applicability of these hydrogels as scaffolds for cell culturing [25].

### Swelling kinetics of PASP gels in the presence of DTT

**Figure 3:**
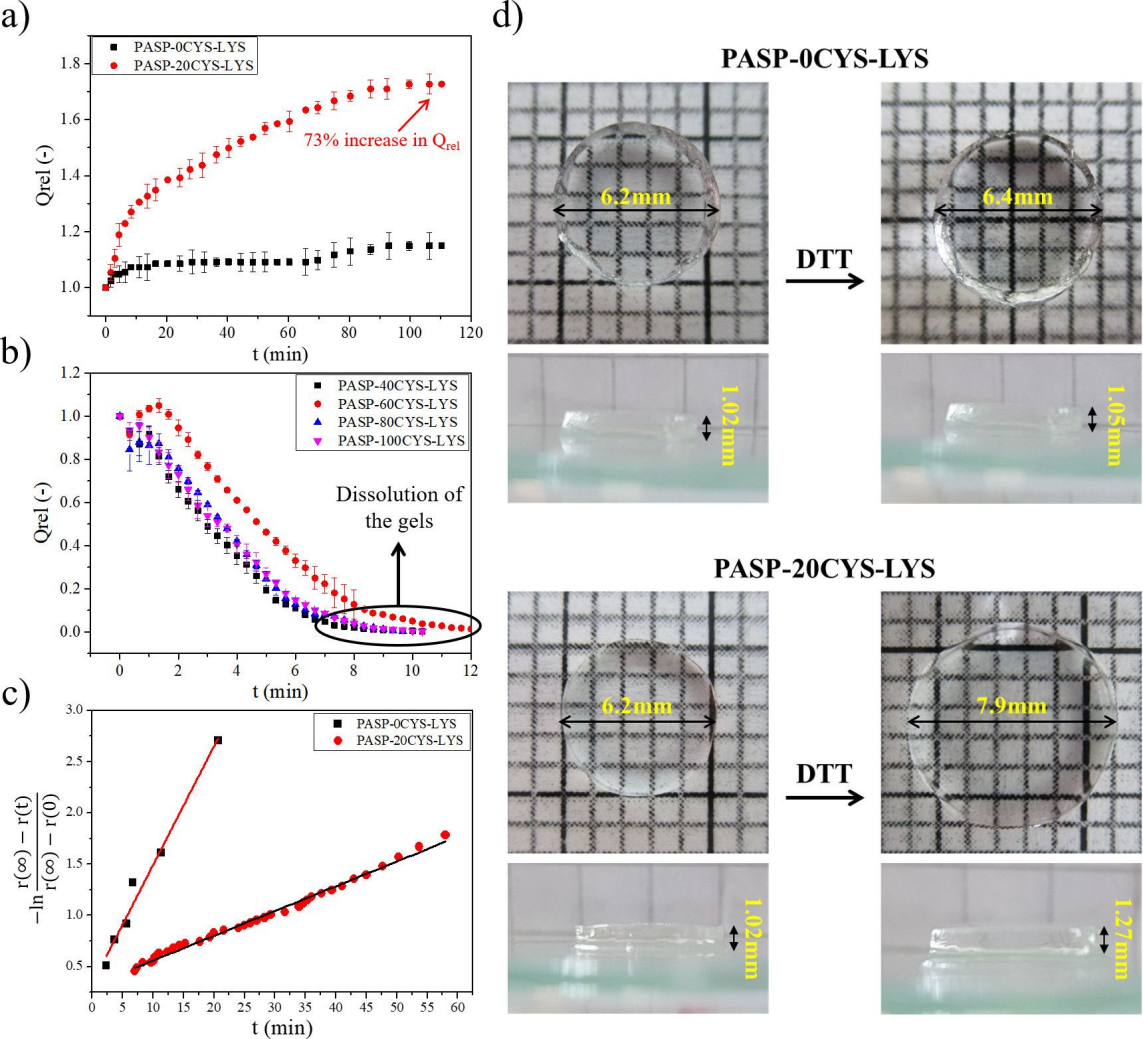
(a) Swelling and (b) dissolution kinetics of different PASP-XCYS-LYS gels in 0.1 M DTT solution at pH 8. (c) Description of the swelling kinetics by THB theory [12] and (d) images of the gels before and after swelling.

The disulfide bonds in the PASP-based hydrogels are sensitive to the redox potential of the local environment [21,34,45]. Therefore, in the presence of a reductive agent such as DTT, the disulfide bonds can be cleaved and the gel can swell in the same way as it can happen under physiological conditions or in vitro. In this section, we demonstrate the results of the swelling kinetics in the presence of DTT (Figure 3).

Figure 3a and Figure 3b show the changes of the relative volume degree of the swelling (*Q*_rel_) of the gels dissolved in 0.1 M DTT at pH 8. The 0CYS-LYS gel is not sensitive to the DTT solvent because it does not contain any disulfide bonds. Due to the polyelectrolyte character of PASP, the small but fast increase of *Q*_rel_ for PASP-0CYS-LYS is most likely caused by the small change of the ionic concentration of the solution (Figure 3a and Figure 3d) [52]. The swelling degree of the 20CYS-LYS gel increases significantly (73%) in the presence of DTT within 2 h, which proves the cleavage of the disulfide bonds in the CYS cross-linkers. Due to the higher cross-linking ability of CYS compared to LYS, *Q*_rel_ increases almost by a factor of two in these gels although CYS represents only 20% of the total cross-linker molecules. For other permanent cross-linkers, such as DAB [21] or poly(ethylene glycol) diglycidyl ether [34], the reduction of the disulfide bonds leads to a lower swelling degree, which also proves the previously mentioned theory. Upon increasing the density of the CYS molecules further, the gels abruptly degrade and dissolve after a few minutes (8–10 min), since the cross-linking density reaches the critical value at which the osmotic pressure bursts the polymer matrix [53].

To describe the swelling kinetics of the 0CYS-LYS and 20CYS-LYS samples, the TFPC theory was used as has been shown in our previous article [13] (Table 2).

**Table 2:** Swelling kinetics data of the different gels.

Sample	Final *Q*_rel_	*r*(0) (mm)	*r*(∞) (mm)	τ (min)

0CYS-LYS	1.09	5.95	6.24	8.69 ± 0.7
20CYS-LYS	1.72	5.95	7.15	41.67 ± 2.1

The swelling of the two hydrogels is based on different processes. The swelling of the 0CYS-LYS hydrogel occurs due to changes of the ionic strength, while the swelling of the 20CYS-LYS hydrogel is induced by the cleavage of the disulfide bonds. The change of the ionic strength is a faster process, therefore, the relaxation time determined for the 0CYS-LYS hydrogel is smaller than that of the 20CYS-LYS hydrogel.

### Dependence of the swelling degree of the PASP-20CYS-LYS gels on the amount of DTT in the environment

**Figure 4:**
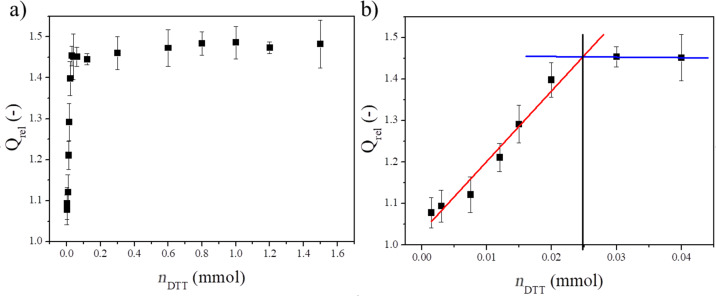
Dependence of the relative swelling degree of the PASP-20CYS-LYS gel on the amount of DTT a) between 0 and 1.6 mmol DDT and b) between 0 and 0.04 mmol DTT.

In order to investigate whether every disulfide bond in the gel disks has been cleaved in the previous experiment and to get a better view of the fate of the scaffold during possible future therapeutic applications, the changes of the swelling degree were measured in the presence of different amounts of DTT. The amount of disulfide bonds was determined in the PASP-20CYS-LYS gels (Figure 4).

The relative swelling degree (*Q*_rel_) shows a monotonic increase with the increasing amount of DTT up to about 0.03 mmol (Figure 4). Elevating the amount of DTT, no further changes in *Q*_rel_ can be seen. In order to determine the amount of disulfide bridges in the gel, a linear regression line was fitted to the first part of the measurement points (0−0.03 mmol DTT) and the constant part (0.03−1.6 mmol DTT) (Figure 4b). The *x*-coordinate of the intersection point of the two lines gives the minimum amount of DTT (0.025 mmol) that can cleave the total amount of the disulfide bridges. Hence, this amount it is equal to the molar amount of the disulfide bonds in the gel matrix. According to these data, the amount of disulfide bonds in the cross-linkers of the PASP-20CYS-LYS hydrogels is around 0.025 mmol, which is equal to the amount of CYS cross-linkers applied in the synthesis (see above). These results prove that each disulfide bond in the PASP-20CYS-LYS hydrogels has been cleaved by DTT in the described experiments (Figure 3). Due to the dissolution of the hydrogels containing higher amounts of CYS cross-linkers, this method cannot be used to determine the amount of disulfide bonds in these gels.

It is a challenging task to determine the cross-linking density in a polymer matrix. Usually, stress–strain measurements are used for this purpose. However, their results are influenced not only by the density of the chemical cross-links but also by the physical interactions between the polymer matrix and the swelling agent as well as other thermodynamic parameters [21,50–51,54–56]. Consequently, the swelling behavior of different hydrogels needs to be investigated in depth, and the relation between the swelling and other thermodynamic parameters is still to be determined. However, such measurements are difficult and require expensive equipment. By the presented method, the number of chemical cross-links can be determined by cleaving the sensitive bonds in the cross-linkers. The changes of the swelling degree as a function of the concentration of the reactant reveal the density of the sensitive bonds. This method is not reported in the literature about polymer gels. Thus, it can be regarded as a novel protocol for the determination of the amount of the sensitive cross-linkers in hydrogels.

### Relationship between the mechanical properties and the chemical constitution of the gels

The stiffness of the hydrogel scaffold is a key parameter in the field of tissue engineering. It was described previously that different cell types prefer gels of different stiffness for proliferation [57]. In this section, the mechanical properties of the amino acid-based hydrogels of different chemical constitutions will be explained in detail.

**Figure 5:**
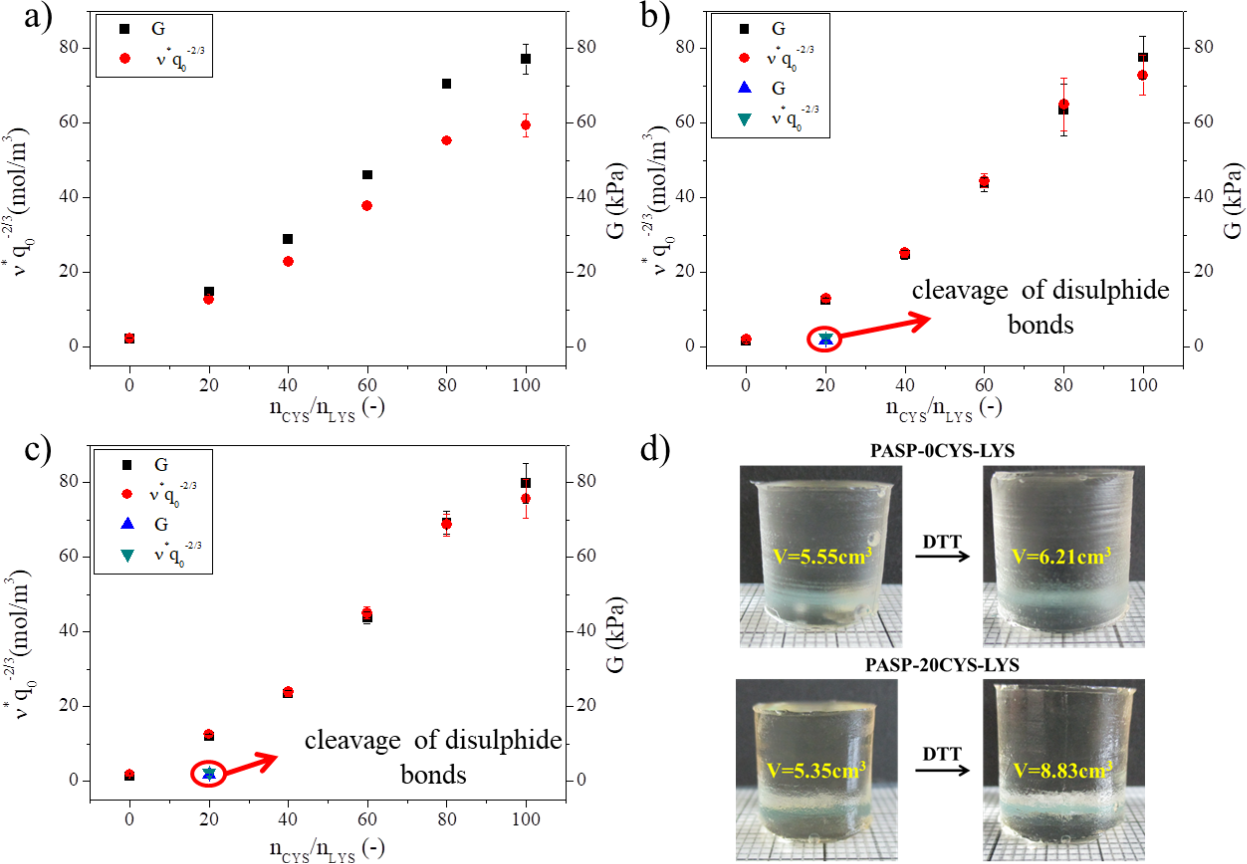
Dependence of the elastic modulus *G* (black squares, blue triangles) and the concentration of the elastically active chains v*q_0_^−2/3^ (red dots, green triangles) on the CYS/LYS molar ratio in a) DMSO, b) imidazole buffer (pH 8, *c* = 0.1 M, *I* = 0.25 M) and c) PBS (pH 7.4, *I* = 0.15 M). d) Gel cylinders before and after DTT treatment.

As shown in Figure 3, the swelling properties of the PASP-based gels significantly depend on the chemical constitution of the gels. Consequently, the mechanical properties presumably depend on it as well. The relationship between the molar ratio of the two cross-linking agents (*n*_CYS_/*n*_LYS_) and the mechanical properties such as the elastic modulus (*G*) and the concentration of the elastically active chains (v*q_0_^–2/3^) can be seen in Figure 5.

Upon increasing the CYS/LYS molar ratio, both *G* and ν* monotonically increase in every solvent (Figure 5). This linear increase provides evidence that CYS has a higher cross-linking ability than LYS during the gel synthesis. Namely, larger amounts of cross-links in the polymer matrix lead to higher values of *G* and ν* [21]. This indicates that a larger amount of CYS molecules reacts with two amine groups cross-linking the polymer chains. The strong electron withdrawing character of the carboxyl group of the amino-acid group decreases the electron density of the alpha amine group, resulting in a weaker reactivity of LYS. Supposedly, most LYS molecules react only with their side chain amine groups, and the gelation time is longer if the ratio of LYS cross-linkers is higher. This behavior can also contribute to the characteristic dependence of the gel swelling on the pH value (see above). The cross-linking ability of LYS can be improved by phosphoric acid catalysis as was shown by Gyenes and co-workers [22]. However, this method cannot be used in our case because phosphoric acid precipitates CYS immediately.

Upon hydrolysis, the ν* values increase only very slightly, while the *G* values decrease (Figure 5a and Figure 5b). Neither of these two parameters changes when replacing the imidazole buffer (pH 8) with PBS (Figure 5b and Figure 5c). The ν* values depend on the swelling degree of the gels (Equation 2), which is changing during hydrolysis (Supporting Information File 1, Table S1). Yet, the swelling degree is not influenced by the change of the buffer from imidazole to PBS since the concentration of ions remains the same in both buffer solutions. This may explain the different values of *G* and ν* in DMSO (Figure 5a) but both values are similar after hydrolysis. These findings are in accordance with our previous findings [21].

When cleaving the disulfide bonds using DTT, all gels except for the 20CYS-LYS gel turned very soft or dissolved completely (Supporting Information File 1, Figure S2). However, the 20CYS-LYS gel did not dissolve (Figure 5d) but only swelled and turned moderately softer after the DTT treatment. The values of *G* and ν* decreased significantly (blue and green triangles in Figure 5b and Figure 5c). A similar decrease has been observed for other PASP-based CYS containing hydrogels in which another type of nonsensitive cross-linker was applied simultaneously with CYS [21,34,45].

### Application of the PASP-XCYS(E)-LYS gels for cell cultivation

In addition to the elaborate characterization of the swelling and the mechanical properties as well as the thiol content, our aim was to study the applicability of the amino acid-based hydrogels as scaffolds for cell cultivation.

**Figure 6:**
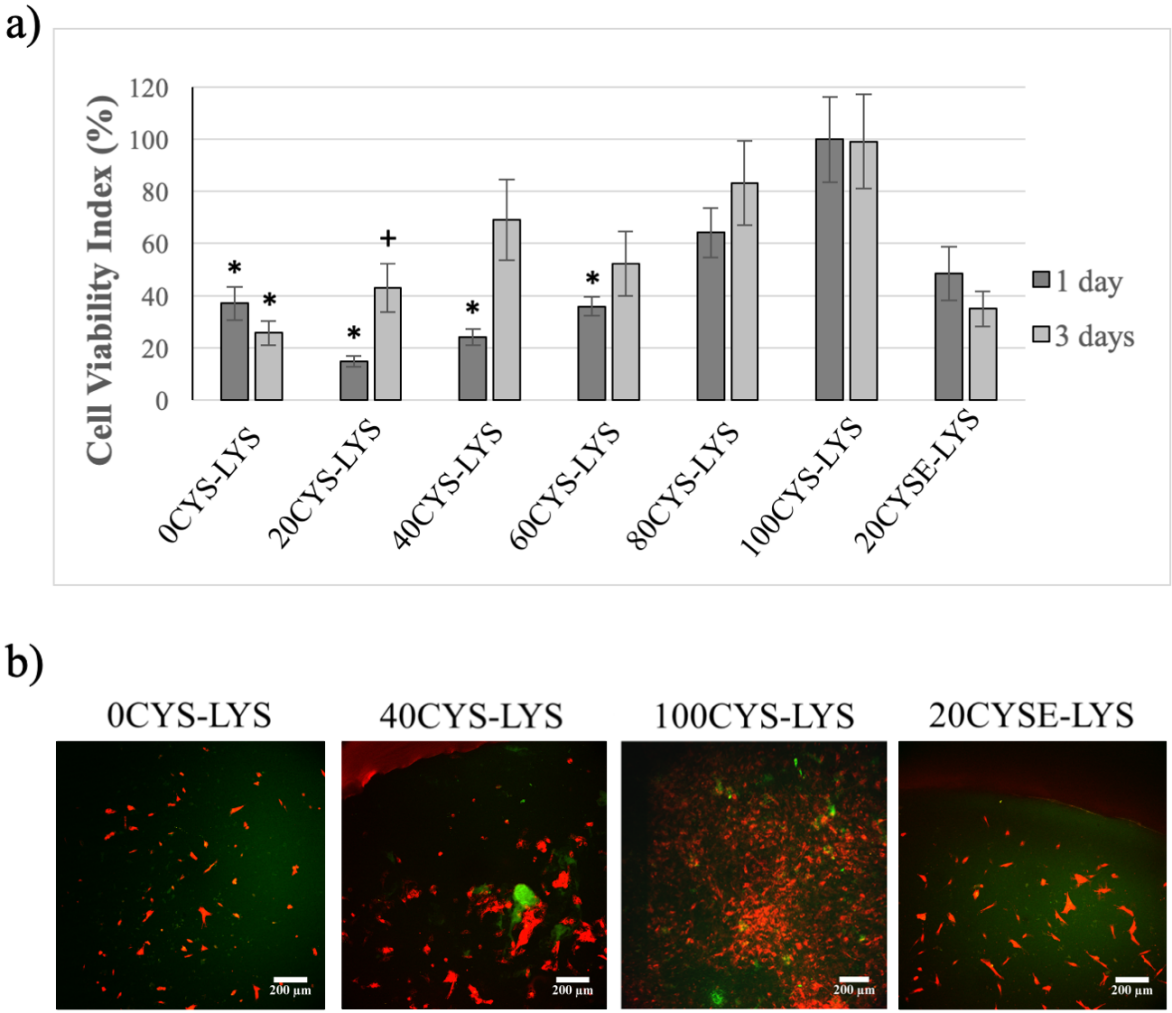
a) Viability of the PDLCs measured one and three days after seeding. The average viability value measured on the 100CYS-LYS gel on day one was considered as 100%. The cell viability indices are given as arithmetic mean ± SEM. * indicates p < 0.05 compared to the value of 100CYS-LYS at the corresponding time. + indicates p < 0.05 compared to the value of 100CYS-LYS on day one. b) Two-photon microscopic images of the PDLCs on the different hydrogels after three days. The scale bar indicates 200 μm. Each photomicrograph was taken at the same magnification.

At first, the viability of the growth of PDLCs on the different hydrogels was tested (Figure 6a). After one day of cell growth, the highest cell viability index (referring to the highest cell adhesion) was measured on the 100CYS-LYS gel, which does not contain any LYS at all, only the redox sensitive cross-linker CYS. On day one, the cell viability index was lower for the 20CYS-LYS gel and the 40CYS-LYS gel than for the 100CYS-LYS gel. Still, for both gels, it had doubled after three days of cell growth suggesting that a lower CYS concentration favors cell proliferation. This result is presumably related to the enhanced elastic modulus of the hydrogels (see above) as well as the relatively low LYS concentration. These results are in accordance with our previous work where we demonstrated that exchanging half of the CYS cross-linkers by DAB improved the proliferation of osteosarcoma cells [25]. However, the aim of the present study is to prepare gels composed of amino acids exclusively and test their biocompatibility on healthy cells. The disulfide bonds in the polymer matrix were supposedly cleaved in the cell medium during the three days of incubation which results in the formation of thiol groups in the hydrogel. The presence of thiol groups supports cell adhesion and proliferation as we showed previously. Therefore, the higher the density of CYS in the polymer matrix, the higher is the viability of the cells [25]. In the literature, several articles can be found about the effect of poly-ʟ-lysine on cell adhesion and proliferation [30,58–59]. It was elucidated that this advantageous feature of poly-ʟ-lysine is concentration-dependent, and the amount of poly-ʟ-lysine should be optimized to reach the highest cell viability [30]. In our hydrogels, LYS is not polymerized but present as a monomer and plays a role as a cross-linker. This function of LYS in scaffolds, which is related to cell proliferation, has not been investigated before. Nevertheless, the positive effect of LYS is highly concentration-dependent. According to the literature, a high amount of LYS has a cytotoxic effect, while a lower amount supports cell adhesion and proliferation. Moreover, the effect of LYS can be diverse depending on whether poly-ʟ-lysine is applied in water-soluble form [58] or as a built-in element in scaffolds [30]. On the other hand, LYS is a permanent cross-linker which is important to maintain the stability of the hydrogel during the cell experiments. Based on our results (Figure 6), the highest viability increase from day one to day three was found for the 20CYS-LYS and 40CYS-LYS gels, suggesting that cross-linker ratios of 60−80% LYS and 20−40% CYS yield the highest proliferation rate of PDLCs. The viability of cells on the 0CYS-LYS gel as well as on the 20CYSE-LYS gel, for which the disulfide bonds were previously created using the reductive agent DTT, decreased slightly from day one to day three. The similar behavior of the cells on these two hydrogels can be explained by the similar gel structure. Neither of these two gels contains CYS as a cross-linker, but only LYS cross-links the polymer chains. As given in Table 1, the initial concentration of LYS in our gels was between 0 and 1.8 wt %. A significant increase of the cell viability from day one to day three was measured for the 40CYS-LYS gels (LYS concentration around 1.5 wt %) which correlates with the findings of Datta et al., who found that the addition of 1.5 wt % of LYS significantly increases the cell viability [30].

The two-photon microscopic images (Figure 6b) confirm the results of our viability assays. Since the cells show a red fluorescence due to the vital stain applied, while the PASP-based hydrogels show a green autofluorescence [25], the gel matrix and the cells are easily distinguished. On day three, only few PDLCs showed a healthy fibroblast morphology on the 0CYS-LYS hydrogel, while the other cells had a rounded shape suggesting that they were unable to adhere to these gels. By increasing the ratio of the CYS cross-linking agents in the gel matrix, the proportion of the viable cells became elevated. The highest amount of PDLCs with fibroblast morphology can be observed on the 100CYS-LYS gel, which correlates well with the viability results.

According to the literature, various forms of lysine-containing scaffolds are able to enhance both the adhesion and the proliferation of stem cells. The most commonly used scaffolds are based on poly-ʟ-lysine [30,59–60] and poly-ᴅ-lysine [61]. However, LYS was applied as a cross-linker only in a few cases. The corresponding gels are not fully composed of amino acids or have other disadvantages compared to our gels, for example, expensive preparation methods [62–63].

### Drug release kinetics in isotonic and reductive medium

In addition to the possible applications in tissue engineering, the applicability of the amino acid-based hydrogels in drug delivery is very important. Therefore, the second aim of our work was to investigate the potential of the scaffolds as drug carriers. To get a better understanding of the fate of the drug in the living system, the release kinetics were determined not only in an isotonic but also in a reductive medium. Metoprolol was chosen as a model drug because it does not chemically interact with the polymer backbone, but ionic interactions can occur between the polymer matrix and the metoprolol molecules. Hence, theoretically, the only limiting factor of the release of metoprolol is diffusion. Moreover, metoprolol can be measured easily beside DTT by UV–vis spectroscopy.

**Figure 7:**
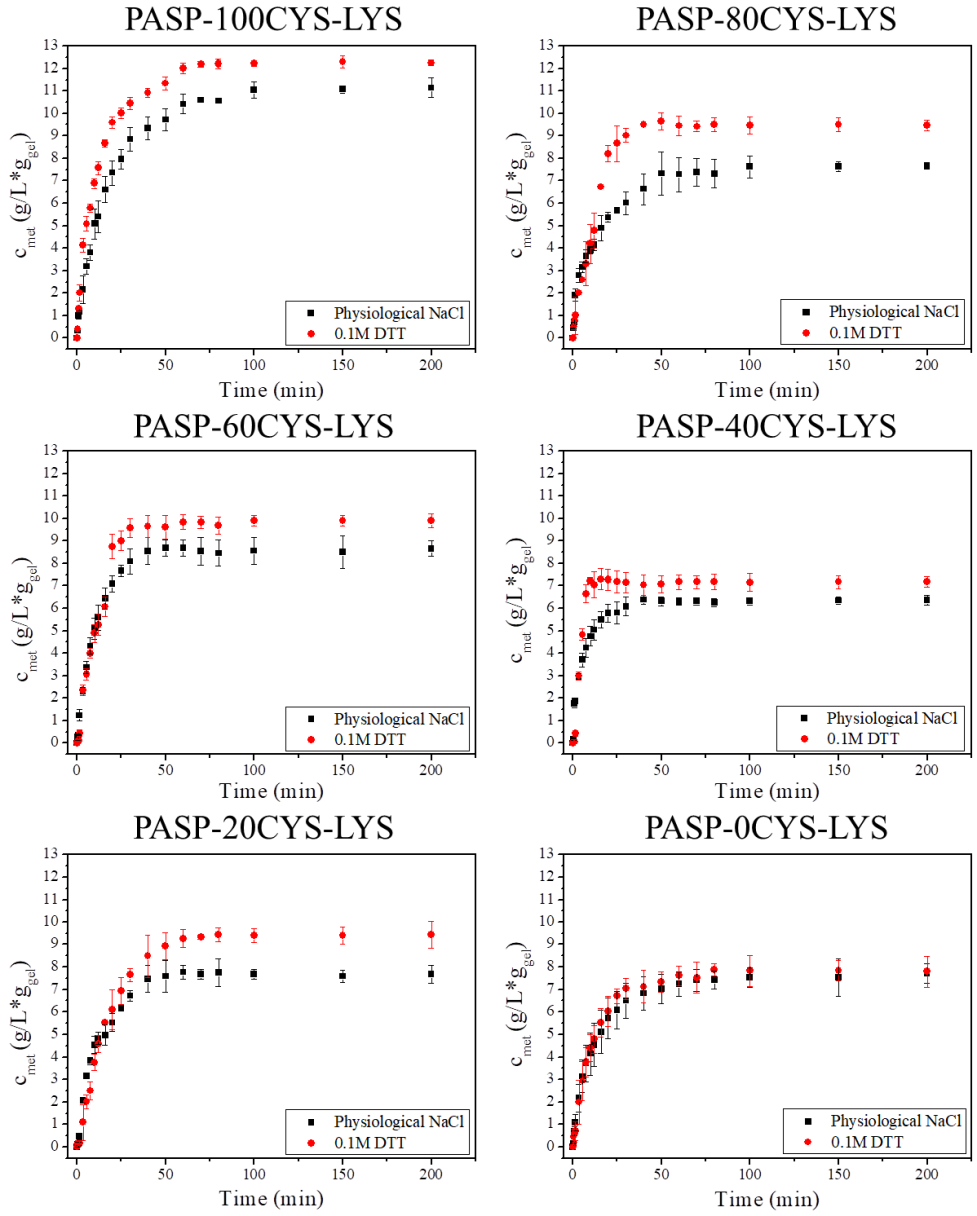
Metoprolol release from the different PASP-XCYS-LYS gels (*c*_met_) in physiological saline solution (black squares) and in 0.1 M DTT/physiological saline solution (red circles).

The kinetics of the metoprolol release from the different PASP-XCYS-LYS gels was studied in both physiological saline (NaCl) and DTT solution (Figure 7). The graph depicts the released concentration of metoprolol normalized to the initial mass of the gel disk (*c*_met_) as a function of release time.

The drug release kinetics show a similar behavior for all gel samples in the physiological saline solution (Figure 7, black squares). The released metoprolol reaches the saturation concentration after ca. 50 min in every measurement. The saturation concentration is highest for the PASP-100CYS-LYS sample reaching a concentration of 11 g/L∙g_gel_ in physiological NaCl. Upon decreasing the density of CYS cross-links in the hydrogels, the saturation concentration is reduced to around 7.5 g/L∙g_gel_ for the 20CYS-LYS and the 0CYS-LYS hydrogel. A possible explanation of this phenomenon is the following. Metoprolol has a secondary amino group, which is protonated under these conditions leading to a weak interaction with the negatively charged PASP. As we demonstrated in previous experiments, hydrogels with increasing density of CYS cross-linkers have a higher polymer content (higher elastic modulus). Therefore, gels of higher CYS content can take up more metoprolol molecules from the solution. Consequently, a higher amount of CYS in the gel can result in a higher saturation concentration of metoprolol in the liquid. The kinetics curves are very similar to normal kinetics curves of drug release indicating a burst effect [15].

The presence of DTT leads to a higher saturation concentration of metoprolol presumably due to the cleavage of the disulfide bonds. The dissolution of the PASP-100CYS-LYS gel in the reductive medium is a continuous process. Therefore, the concentration of the released metoprolol in the DTT solution was higher than in the physiological salt solution at each measurement point. For the 40CYS-LYS, the 60CYS-LYS and the 80CYS-LYS gels, the metoprolol concentration increases faster compared to the 0CYS-LYS and the 20CYS-LYS gels in the first 20 min of the metoprolol release, which is presumably caused by the dissolution of the gels (Figure 3b). Although the dissolution of the 40CYS-LYS, the 60CYS-LYS, the 80CYS-LYS and the 100CYS-LYS gels occurs already after 10 min in DTT solution (Figure 3b), the concentration of metoprolol reaches the saturation concentration only after 20 min. The ionic interaction between the polymer backbone and metoprolol could slow down the dissolution of the gels, which can cause such a difference. A higher degree of swelling leads to a higher diffusion coefficient in the gel matrix and consequently to a faster drug release. A similar observation was described in the case of another PASP-based gel and different other types of hydrogels [34]. For the PASP-20CYS-LYS gel, the drug release kinetics do not differ significantly for the reductive medium and the physiological solution during the first 20 min of release. Thus, the cleavage of the disulfide bonds in PASP-20CYS-LYS seems to be slower. After 20 min, this gel already swelled significantly and the metoprolol concentration increased in DTT solution. The saturated concentration reached a higher value in the DTT solution than in the physiological saline solution by the end of the measurement. The observation that DTT had no influence on the drug release in the case of 0CYS-LYS (Figure 7) is in line with the findings of our study of the swelling kinetics, i.e., that this gel type lacks disulfide bonds making it insensitive to the reductive environment (Figure 3a).

## Conclusion

In this work, we tested the suitability of different fully amino acid-based hydrogels for tissue engineering and drug delivery applications. The influence of the chemical constitution of these hydrogels on the swelling and the mechanical properties was studied under different conditions. To prepare PASP-based hydrogels, LYS and CYS were used as cross-linkers at different molar ratios. Whereas LYS acts as a permanent cross-linker in the polymer matrix, CYS is sensitive to changes of the redox potential in the environment. Thus, the redox response of the hydrogels significantly depends on the ratio of the two cross-linkers. By increasing the CYS content, the swelling of the hydrogels becomes faster in different media. According to the mechanical tests, LYS has a lower cross-linking ability than CYS due to the ester group in LYS (lysine methylester). Therefore, a higher LYS content results in a lower elastic modulus and a lower concentration of elastically active chains in the hydrogels. The cleavage of the disulfide bonds leads to a significant decrease of the concentration of the elastically active chains for the 20CYS-LYS gel, which highly influences the release of metoprolol from the gel. Using different amounts of DTT as a reducing agent, the amount of CYS as the reactive cross-linker could be determined precisely. Human PDLCs were used to assess the biocompatibility of the hydrogels. Their viability also proved to depend on the ratio of the two cross-linkers. In summary, PASP-based hydrogels are promising materials for both medical and pharmaceutical applications which can be designed by tailoring the chemical structure of the hydrogels.

## Supporting Information

File 1 Additional experimental information.
