# Supplementary material for: Fully amino acid-based hydrogel as potential scaffold for cell culturing and drug delivery
Source: Beilstein J Nanotechnol. 2019 Dec 27;10:2579–93. doi: 10.3762/bjnano.10.249 (PMC6941446; doi:10.3762/bjnano.10.249)
Supplement: File 1 — Additional experimental information. [file Beilstein_J_Nanotechnol-10-2579-s001.pdf]

BEILSTEIN JOURNAL OF NANOTECHNOLOGY

Supporting Information

for

Fully amino acid-based hydrogel as potential scaffold for cell culturing and drug delivery

Dávid Juriga, Evelin Sipos, Orsolya Hegedűs, Gábor Varga, Miklós Zrínyi, Krisztina S. Nagy and Angéla Jedlovszky-Hajdú

Beilstein J. Nanotechnol. **2019,** *10,* 2579–2593. doi:10.3762/bjnano.10.249

Additional experimental information

License and Terms: This is a supporting information file under the terms of the Creative Commons Attribution License (https://creativecommons.org/licenses/by/4.0). Please note that the reuse, redistribution and reproduction in particular requires that the authors and source are credited.
The license is subject to the *Beilstein Journal of Nanotechnology* terms and conditions: (https://www.beilstein-journals.org/bjnano)

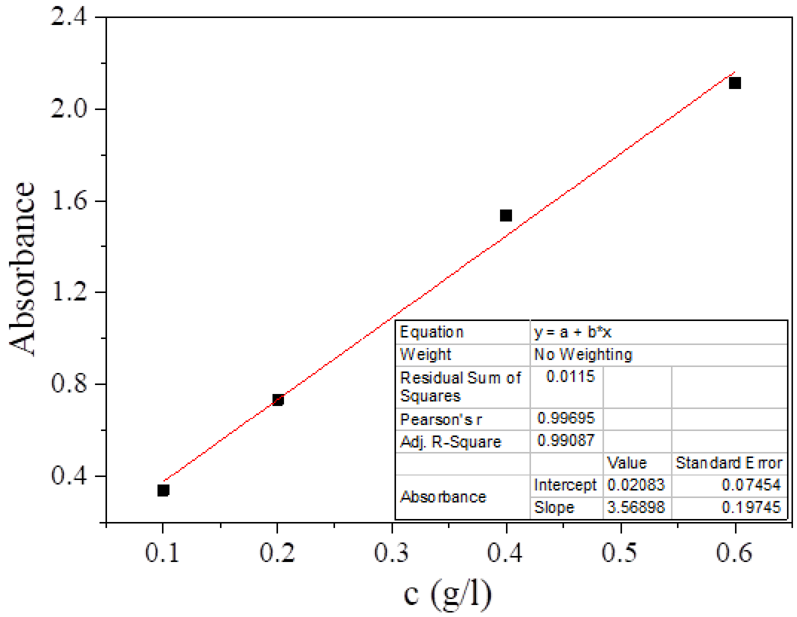

Figure S1: Metropolol calibration line in physiological saline solution for release measurement.

Table S1: Elastic modulus and concentration of elastically active chains of XCYS-LYS gels in different swelling agent. Red numbers nominate the elastic modulus and concentration of elastically active chains after DTT treatment

Gel		DMSO (PSI form)	pH 8 (PASP form)	PBS (PASP form)
100CYS-LYS	G (kPa)	73.42 ± 7.50	74.85 ± 5.71	75.90 ± 0.09
	$v^* q_0^{-2/3}$ (mol/m^3)	50.91 ± 5.20	69.66 ± 5.31	72.05 ± 0.09
80CYS-LYS	G (kPa)	70.43 ± 1.35	63.45 ± 6.95	69.23 ± 3.04
	$v^* q_0^{-2/3}$ (mol/m^3)	55.28 ± 1.06	64.96 ± 7.11	68.58 ± 3.01
60CYS-LYS	G (kPa)	46.08 ± 0.71	43.63 ± 1.88	43.82 ± 1.55
	$v^* q_0^{-2/3}$ (mol/m^3)	37.80 ± 0.58	44.55 ± 1.91	45.05 ± 1.59
40CYS-LYS	G (kPa)	28.81 ± 0.97	24.69 ± 1.15	23.36 ± 1.02
	$v^* q_0^{-2/3}$ (mol/m^3)	22.82 ± 0.77	25.15 ± 1.17	23.86 ± 1.04
20CYS-LYS	G (kPa)	14.80 ± 0.99	12.43 ± 0.72<br>1.79	11.86 ± 0.73<br>1.68
	$v^* q_0^{-2/3}$ (mol/m^3)	12.61 ± 0.84	12.91 ± 0.75<br>2.42	12.54 ± 0.77<br>2.35
0CYS-LYS	G (kPa)	2.22 ± 0.32	1.48 ± 0.23<br>1.48	1.26 ± 0.63<br>1.37
	$v^* q_0^{-2/3}$ (mol/m^3)	2.28 ± 0.33	1.97 ± 0.31<br>2.03	1.91 ± 0.96<br>1.93

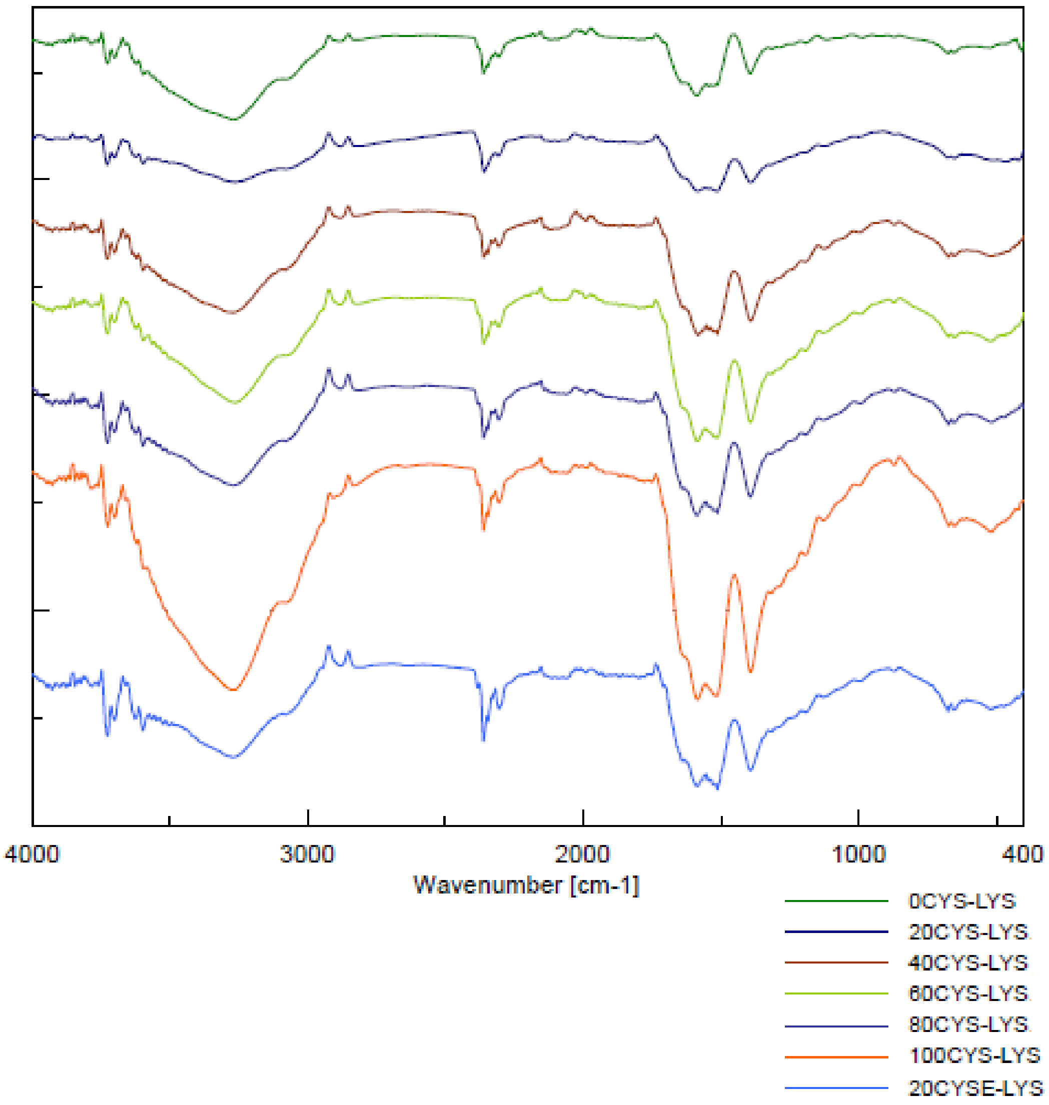

Figure S2: FTIR spectra of different hydrogels. The chemical composition of the gels was investigated via Fourier transform infrared spectroscopy (FTIR) using a JASCO FT/IR-4700 spectrophotometer fitted with an attenuated total reflection (ATR) accessory (JASCO ATR Pro One). The spectra were collected in a range of (400–4000 cm^{-1}) with a resolution of 2 cm^{-1} and a scan number of 126. The baseline of the spectra was corrected using the JASCO spectra analysis program. As seen in the Figure, there is no relevant difference between the chemical structures of the hydrogels. The wide peak above 3000 cm^{-1} is connected to the carboxyl group of the PASP, while the $\nu_{C=O}$ and $\nu_{C\text{-}N}$ stretching modes of amide group appear at 1660 and 1510 cm^{-1} which signals are proving the successful hydrolysis of PSI gels. Since there are no specific bands of the succinimide rings on the spectra, all of the rings are most likely successfully opened and hydrolyzed to aspartic acid. The change in the ratio of the two cross-linkers cannot be proved by FTIR spectroscopy because the cross-linking density was low in the samples, and consequently, the signal of the specific peaks of the cross-linker molecules is very weak.

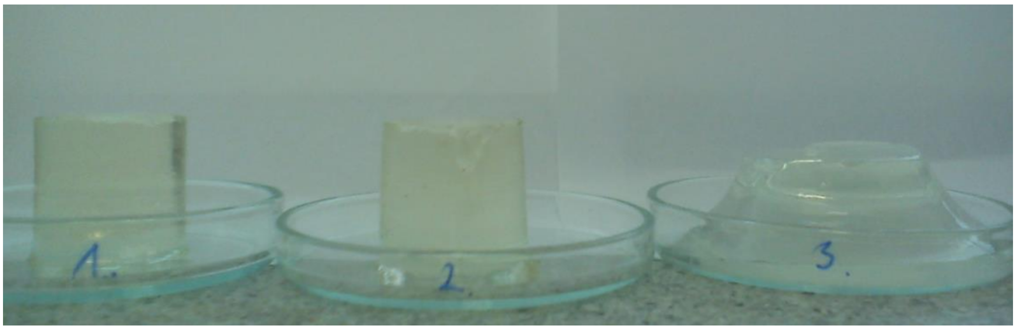

Figure S2: Photographs of the different PASP gel cylinders after DTT treatment. 1) PASP-0CYS-LYS, 2) PASP-20CYS-LYS, 3) PASP-40CYS-LYS
